# Protection of bats *(Eptesicus fuscus)* against rabies following topical or oronasal exposure to a recombinant raccoon poxvirus vaccine

**DOI:** 10.1371/journal.pntd.0005958

**Published:** 2017-10-04

**Authors:** Ben Stading, James A. Ellison, William C. Carson, Panayampalli Subbian Satheshkumar, Tonie E. Rocke, Jorge E. Osorio

**Affiliations:** 1 Department of Pathobiological Sciences, University of Wisconsin - Madison, Madison, Wisconsin, United States of America; 2 Poxvirus and Rabies Branch, Division of High-Consequence Pathogens and Pathology, National Center for Emerging Zoonotic Infectious Diseases, Centers for Disease Control and Prevention, Atlanta, Georgia, United States of America; 3 US Geological Survey, National Wildlife Health Center, Madison, Wisconsin, United States of America; Wistar Institute, UNITED STATES

## Abstract

Rabies is an ancient neglected tropical disease that causes tens of thousands of human deaths and millions of cattle deaths annually. In order to develop a new vaccine for potential use in bats, a reservoir of rabies infection for humans and animals alike, an *in silico* antigen designer tool was used to create a mosaic glycoprotein (MoG) gene using available sequences from the rabies Phylogroup I glycoprotein. This sequence, which represents strains more likely to occur in bats, was cloned into raccoonpox virus (RCN) and the efficacy of this novel RCN-MoG vaccine was compared to RCN-G that expresses the glycoprotein gene from CVS-11 rabies or luciferase (RCN-*luc*, negative control) in mice and big brown bats (*Eptesicus fuscus*). Mice vaccinated and boosted intradermally with 1 x 10^7^ plaque forming units (PFU) of each RCN-rabies vaccine construct developed neutralizing antibodies and survived at significantly higher rates than controls. No significant difference in antibody titers or survival was noted between rabies-vaccinated groups. Bats were vaccinated either oronasally (RCN-G, RCN-MoG) with 5x10^7^ PFU or by topical application in glycerin jelly (RCN-MoG, dose 2x10^8^ PFU), boosted (same dose and route) at 46 days post vaccination (dpv), and then challenged with wild-type big brown variant RABV at 65 dpv. Prior to challenge, 90% of RCN-G and 75% of RCN-MoG oronasally vaccinated bats had detectable levels of serum rabies neutralizing antibodies. Bats from the RCN-*luc* and topically vaccinated RCN-MoG groups did not have measurable antibody responses. The RCN-rabies constructs were highly protective and not significantly different from each other. RCN-MoG provided 100% protection (n = 9) when delivered oronasally and 83% protection (n = 6) when delivered topically; protection provided by the RCN-G construct was 70% (n = 10). All rabies-vaccinated bats survived at a significantly (P ≤ 0.02) higher rate than control bats (12%; n = 8). We have demonstrated the efficacy of a novel, *in silico* designed rabies MoG antigen that conferred protection from rabies challenge in mice and big brown bats in laboratory studies. With further development, topical or oronasal administration of the RCN-MoG vaccine could potentially mitigate rabies in wild bat populations, reducing spillover of this deadly disease into humans, domestic mammals, and other wildlife.

## Author summary

Rabies remains a significant and costly zoonotic disease worldwide. While control of canine rabies can significantly diminish the threat to human health, spillover of rabies and related lyssaviruses from bats into terrestrial animals and humans continues to be an important issue. Here we describe the development of a novel rabies vaccine, using raccoonpox virus (RCN) as a viral vector, and a computer designed rabies virus mosaic antigen. We demonstrate that this new vaccine leads to protection against experimental challenge in wild caught big brown bats when administered oronasally or topically. This technology could be adapted to target other bat species and also be directly applicable toward control of vampire-bat associated rabies in Mexico and Central and South America.

## Introduction

Rabies is a fatal viral zoonotic disease known to humans for nearly four millennia that continues to cause significant public health concern with over 50,000 human deaths every year [1]. Fortunately, over 15 million people receive post-exposure prophylaxis for rabies exposure, which effectively prevents rabies if administered promptly [2]. In Mexico and Central and South America, rabies transmitted by vampire bats is a tremendous public health and economic issue, as it threatens not only the people in these areas, but also an at-risk population of more than 70 million head of cattle [3–6]. Vampire bats were thought to have caused cattle losses in Latin America worth more than $40 million US in 1983, and again in 1984 [7], and these losses, coupled with the cost of measures to prevent bovine rabies, are a significant economic burden.

Rabies virus (RABV, Family: *Rhabdoviridae*, Genus: *Lyssavirus*) has adapted to numerous mammalian reservoirs that maintain transmission, typically by bite, and as a result has evolved into specific lineages and variants. Bats are considered the primary evolutionary host of RABV [8] and harbor a diversity of other lyssaviruses, all of which cause rabies disease, with non-RABV lyssaviruses occurring in the Old World and Australia [9,10]. Lyssaviruses are divided into distinct phylogroups based on serological analysis and genome sequence [11]. While lyssaviruses within phylogroup I (PG-I) are considered cross-protective immunologically, epidemiologically important antigenic variation between vaccine strains and wild-type rabies viruses have been observed [12] and variable vaccine efficacy has been reported against some PG-I viruses[13]. In addition, numerous antigenic variants of rabies have been found in bats in the Americas [14]. In Brazil, nine different variants have been reported; in Mexico, at least 7, and antigenic variants differ between bats species and geographic locations.

Rabies in terrestrial wild mammals can be successfully controlled, and in some areas, eliminated through the use of oral rabies vaccination (ORV) campaigns [15–17], but similar mass vaccination has not yet been attempted for wild bats. Recombinant viral-vectored vaccines have been developed to make use of the antigenicity of the RABV surface glycoprotein (G). The main benefit of these viral-vectored constructs is their ability to induce immunity when given orally, which makes them effective and efficient for vaccinating wildlife. A vaccinia virus construct expressing the G protein (or V-RG) has been used extensively for wild carnivores, but this construct can cause vaccinia infection in humans that are inadvertently exposed to the vaccine, especially in immuno-compromised individuals [18–20]. More recently a similar vaccine has been developed and licensed using a human adenovirus vector (ONRAB) [21], but to our knowledge, that vector (and vaccine) has not yet been tested in bats.

Our previous study showed that RCN is a suitable vaccine vector for bats; it safely expressed exogenous antigens and induced significant immune responses following mucosal exposure of *Tadarida brasiliensis* bats [22]. The safety profile of the RCN vector has been evaluated previously [23–25], and a RCN-based sylvatic plague vaccine is under evaluation in field trials in prairie dog populations [26]. In this study, we used G sequences from 664 RABV to design a novel PG-I lyssavirus mosaic glycoprotein gene (MoG) that could potentially provide broader antigenic coverage for the variety of rabies strains circulating in bats, and perhaps a more effective vaccine. We successfully expressed MoG in the RCN vaccine vector and then evaluated its efficacy in preventing rabies mortality in mice and big brown bats (*Eptesicus fuscus*) in laboratory challenge studies, comparing it to a previously reported RCN-G construct that expresses the CVS-11 glycoprotein [27]. Our results suggest that MoG is a successful rabies antigen as both mucosal and topical application of RCN-MoG protected against high-dose rabies virus challenge.

## Methods

### Cells and viruses

Recombinant viruses were generated and amplified on cell monolayers of rat embryonic fibroblasts (Rat-2, ATCC #CRL-1764) or African Green monkey (*Chlorocebus sabaeus)* kidney epithelial cells (BSC40, ATCC #CRL-2761, or Vero, ATCC #CCL-18). Cell cultures were maintained at 37°C and 5% CO_2_ in Dulbecco’s Modified Eagle Medium (DMEM) or Opti-MEM (Life technologies, Madison, WI 53719), supplemented with 2–5% fetal bovine serum (FBS). Recombinant RCN-G [3] and wild-type RCN (RCN-wt) viruses were provided by the Centers for Disease Control (CDC), Atlanta, GA, while the RCN-luc strain used in this study was previously described [28].

The RABV CVS-11 (GenBank accession no AB069973) strain used in mouse challenge studies was provided by the Wisconsin State Laboratory of Hygiene and was amplified on baby hamster kidney cells (BHK-21, ATCC #CCL-10) in DMEM as described elsewhere [29]. The virus was titered by infecting BHK-21 cells in 96-well plates with serial dilutions in quadruplicate. After 72 hours, the cells were fixed with 80% acetone and subsequently probed with a FITC-conjugated rabies antibody (LIGHT DIAGNOSTICS Rabies DFA Reagent 5100, Millipore, Billerica, Massachusetts, USA) to determine focus forming unit (FFU) titer.

The wild type big brown bat variant RABV used for bat challenge has been previously described (GenBank #JQ685920.1); it was isolated from the salivary glands of a naturally infected big brown bat in Pennsylvania during 2006 and subsequently passaged once through murine neuroblastoma cell culture [30]. The virus was provided under a cooperative research and development agreement with the CDC (A06-3684).

### Design and construction of recombinant RCN-MoG virus

#### Design and *in silico* assessment of mosaic rabies glycoprotein

All available sequences for PG-I lyssaviruses (rabies virus, Duvenhage virus, European bat lyssavirus -1 and -2, Aravan virus, Australian bat lyssavirus, Khujand virus, Irkut virus, and Bokeloh bat lyssavirus) were obtained from the National Center for Biotechnology Information (NCBI) and were screened to exclude incomplete and redundant sequences. As a result, a total number of 664 glycoprotein sequences were submitted to the Mosaic Vaccine Designer tool webpage (http://www.hiv.lanl.gov/content/sequence/MOSAIC/makeVaccine.html) to generate a mosaic protein sequence as previously described [31]. Mosaic proteins are assembled *in silico* from fragments of the natural proteins using a genetic algorithm in a way that prevents formation of new epitopes. The program chooses the most frequent epitopes and combines them to form a synthetic antigen, unlike consensus sequences which pick the most frequent amino acid at each position. The parameter options were set as follows: 1) the cocktail size was set to 1 to generate a single peptide that represented all input glycoproteins, 2) the rare threshold was set to 3 for optimal value, and 3) the epitope length parameter was set to an amino acid length of 12-mer in an attempt to match the length of natural T helper cell epitopes. The resulting mosaic lyssavirus glycoprotein was back-translated, codon optimized for expression in vaccinia virus, and then commercially synthesized (GenScript USA Inc., Piscataway, NJ, USA).

Sequences, along with the optimal mosaic vaccine candidate (MoG), were aligned with default settings in muscle v3.8.31 [32] with subsequent manual correction and curation in Mesquite [33]. A maximum likelihood tree was inferred using IQ-TREE v1.4.2 [34] employing the best-fit model of molecular evolution as determined by the automatic model selection procedure (data available upon request). Statistical support values were determined using the ultrafast bootstrap algorithm (n = 1000; [35]) and SH-like approximate likelihood ratio tests (n = 1000; [36]).

#### Construction of recombinant RCN viruses

To aid in the selection of recombinant RCN constructs, we first created an RCN virus with the thymidine kinase (*tk*) gene knocked-out and replaced with green fluorescent protein (GFP). Removal of the *tk* gene results in attenuation of poxviruses without loss of immunogenicity [37], and also serves as a good insertion site for heterologous genes. The RCN-*tk*^*—*^GFP construct was generated by homologous recombination as described elsewhere[38]. Briefly, Vero cells were co-transfected with RCN-*wt*, at a multiplicity of infection (MOI) of 0.05 PFU/cell, and the pTK-GFP plasmid using the FuGENE HD transfection reagent (Promega Corp., Madison, WI, USA). GFP-positive plaques were then selected through 5 rounds of viral purification.

For creating RCN-MoG, the MoG sequence was cloned into the multiple-cloning site (MCS) in the pTK vector under control of the SE/L promoter, and then positive clones were selected. The RCN-MoG construct was then generated by co-transfecting the pTK-SE/L-MoG plasmid and RCN-*tk*^*—*^GFP in BSC-40 cells as described above.

An additional construct was made that utilized an internal ribosomal entry site (IRES) for the expression of the MoG antigen, as it has been found to enhance expression in other constructs [39]. The RCN-IRES-MoG was constructed using the same methods as above by creating a pTK-SE/L-IRES-MoG.

#### *In vitro* expression of RCN-MoG construct

Immunofluorescence and western blot analysis were used to confirm the expression of the artificial MoG antigen by the RCN construct. For immunofluorescence, 6-well plates of Vero cells were infected with RCN-G, RCN-MoG, RCN-IRES-MoG, or RCN-GFP (as a negative control) at an MOI of 1.0 PFU/cell. After 24 hours (h), the plates were fixed with 4% formaldehyde for 10 minutes (min), washed with PBS, then permeabilized with a PBS/0.2%Triton-X-100/0.2% BSA solution for 10 min on ice. The plates were then rinsed and blocked with a PBS/0.02% Triton-X-100/3% BSA solution for 30 min. After blocking, the plates were stained with a 1:1000 dilution of mouse anti-rabies Ab (Rab-50, Invitrogen, Thermo Fisher Scientific Inc., Fitchburg, WI, USA) in blocking solution overnight at 4°C. Primary Ab was then removed, and the wells were washed four times, 10 min each, with a PBS/0.02% Triton-X-100/1.5% BSA washing solution. A secondary Ab solution with a 1:2000 dilution of Alexa Fluor 594 tagged goat anti-mouse Ab (Invitrogen, Thermo Fisher Scientific Inc.) was then added to the wells and left at room temperature for 2 h, followed by an additional four rounds of 10 min washes with the washing solution. Wells were then observed under a fluorescence microscope (excitation wavelength: 590 nm, emission wavelength: 617 nm; AMG EVOS_fl_, Thermo Fisher Scientific Inc.).

For western blot analysis, Vero cells were plated into six-well plates and infected at an MOI of 10 PFU/cell with RCN-MoG, RCN-IRES-MoG, RCN-G, or RCN-*luc* as a negative control. Cells and supernatant were collected 48 h post inoculation and lysed with Laemmli sample buffer (Bio-Rad, Hercules, CA, USA) and heated to 95°C for 5 min. Protein was fractionated via SDS-PAGE and transferred onto a nitrocellulose membrane. Pooled serum from rabies-vaccinated mice (IMRAB3, Merial, Athens, GA, USA) was used as the primary antibody for rabies glycoprotein detection. 3,3’,5,5’-Tetramethylbenzidine (TMB) was used to visualize the glycoprotein in the membranes.

### Animal studies

#### Ethics statement

This study was carried out in strict accordance with recommendations set forth in the National Institutes of Health *Guide for the Care and Use of Laboratory Animals* [40]. All animals and animal facilities were under the control of the School of Veterinary Medicine with oversight from the University of Wisconsin (UW) Research Animal Resource Center. The protocols were approved by the UW Animal Care and Use Committee (approval #’s V01605, V005278), and studies were conducted first in mice, followed by bats.

All animal studies involving rabies virus were conducted under ABSL-2+ conditions in limited access facilities; all individuals involved in the study had documented evidence of pre-existing rabies prophylaxis through recent (<2 years) completion of the full recommended vaccination schedule or confirmation of sufficient circulating rabies neutralizing antibody titers (≥0.5 IU/mL).

#### Mouse challenge study

A/J mice (4 week old) were purchased from Jackson Laboratory (JAX, Sacramento, CA, USA) and housed at the UW Charmany Instructional Facility according to UW husbandry protocols. After a 1 week acclimation period, the mice were separated into 4 treatment groups of n = 16 mice each. Each treatment group was vaccinated with RCN-MoG, RCN-IRES-MoG, RCN-G, or RCN-*luc* via intradermal injection of 1x 10^7^ PFU’s given in 30 μl in the hind limb footpad. Mice were then bled via maxillary lance every 15 days until the rabies challenge, and serum was stored at -80°C. At 75 dpv, mice were boosted with the same vaccine and the same route as previously described. At 208 dpv (133 days post boost), six mice from each group were challenged with 25 x LD_50_ of CVS-11 RABV in 30μl via the intracerebral (IC) route. Mice were then weighed daily and euthanized if they had lost more than 20% of their maximum body weight, or if clinical signs of rabies were evident. All carcasses were frozen at -80°C for diagnostic assessment. The study was ended 14 days after challenge.

#### Bat challenge study

Adult *E*. *fuscus* bats (N = 39) were wild-caught using mist nets in Lee county, Alabama, by Dr. Matthew Grilliot of Troy University under collection permit #8565 provided by the Alabama Department of Conservation and Natural Resources. After acclimation to captive conditions for 4 weeks, the bats were transferred to UW Charmany Instructional facility, where all vaccine studies were conducted. Upon transfer, bats were maintained in screen flight cages (Reptarium, Apogee, Dallas, TX, USA) for a quarantine period of 30 days. During this time blood samples were taken for rabies serology as described below (all bats were negative on intake), and bats were treated topically for parasites with selamectin (Zoetis, Florham Park, NJ, USA). Electronic microchip identification units (Avid Identification Systems, Inc., Folsom, Louisiana, USA) were inserted into each animal, between the scapulae, via subcutaneous injection. Bats were maintained on mealworms (*Tenebrio molitor*), supplemented with vitamins and an omega fatty acids mixture, and water was available *ad libitum*. They were individually weighed at least once per week.

Four bats failed to adapt to captivity and died during quarantine. Two additional bats that continued to lose weight after the quarantine period died 28 days after initial vaccination, and were subsequently tested and found to be rabies negative. The remaining 33 bats formed 4 treatment groups. Three groups of females received 5x10^7^ PFU of RCN-MoG (n = 9), RCN-G (n = 10), or RCN-*luc* (n = 8) via the oronasal (ON) route, with 50μl given orally and 10μl deposited in each nostril (70 μl total volume). One group of males (n = 6) received 2x10^8^ PFU of RCN-MoG mixed with laboratory grade glycerin jelly (Carolina Biological Supply, Burlington, NC, USA) to a final volume of 250μl. This aliquot was distributed equally in the fur of the ventral lateral thorax (near the wing membrane). All bats were anesthetized for inoculation for ~5 minutes and then returned to their cages for recovery. Bats received a booster immunization (same dose and route) at 46 days post initial immunization. All bats were bled via the interfemoral vein on days 0, 21, and 65 dpv. At 65 dpv, bats were challenged with 1x10^5.5^ MICLD_50_/ml of RABV in 100μl delivered bilaterally into the masseter muscles (50μl each). Following challenge, all bats were monitored daily for evidence of disease and weighed twice a week. Any bats that lost ≥20% of their body weight within 7 days or that had evidence of clinical rabies were euthanized under anesthesia by cardiac exsanguination, followed by administration of sodium pentobarbital (Beuthenasia-D, Intervet/Merck Animal Health, Madison, NJ, USA). Carcasses were kept at -80°C until analysis. The study was ended 42 days post challenge, after a 14-day period with no deaths.

#### Rabies diagnosis and serology

Serum rabies neutralizing antibody (RVNA) titers were determined using a microneutralization test that is based on the rapid fluorescent focus inhibition test [40], with some modifications [41]. To determine RVNA titer of individual bats and mice, ten microscopic fields per well on a 4-well slide were scored for presence/absence of at least one fluorescent focus. Endpoint titers were calculated by the Reed-Muench method and were converted to international units (IU/mL) by comparison to a standard rabies immune globulin (SRIG) control containing 2 IU/mL[41]. For the objective of this study, positive RVNA titers (≥0.06 IU/mL) were defined by at least 50% neutralization of the RABV challenge virus dose (50 focus forming doses) at a 1:10 dilution. Final titers less than 0.06 IU/mL were considered negative for the presence of RVNA for the purposes of this investigation.

All mouse and bat carcasses were analyzed for evidence of rabies disease. Brain impressions were fixed in acetone at -20°C, and RABV antigens were detected by the direct fluorescent antibody test (dFA), using fluorescein isothiocyanate (FITC)-labelled monoclonal antibody (mAb) conjugate (Fujirebio Diagnostics, Inc., Malvern, PA, USA) as described [42].

#### Statistical analysis

One-way analysis of variance (ANOVA) was used to analyze neutralizing antibody titers between groups of animals. Wilcoxon matched pairs T-tests were used to compare group body weights over time. Kaplan Meier survival analyses were performed to compare survival between vaccinates and controls. Probability values of 0.05 were considered significant. GraphPad Prism (v6) software (La Jolla, CA, USA) was used for all statistical analyses.

## Results

### Characterization of mosaic constructs

**Fig 1:**
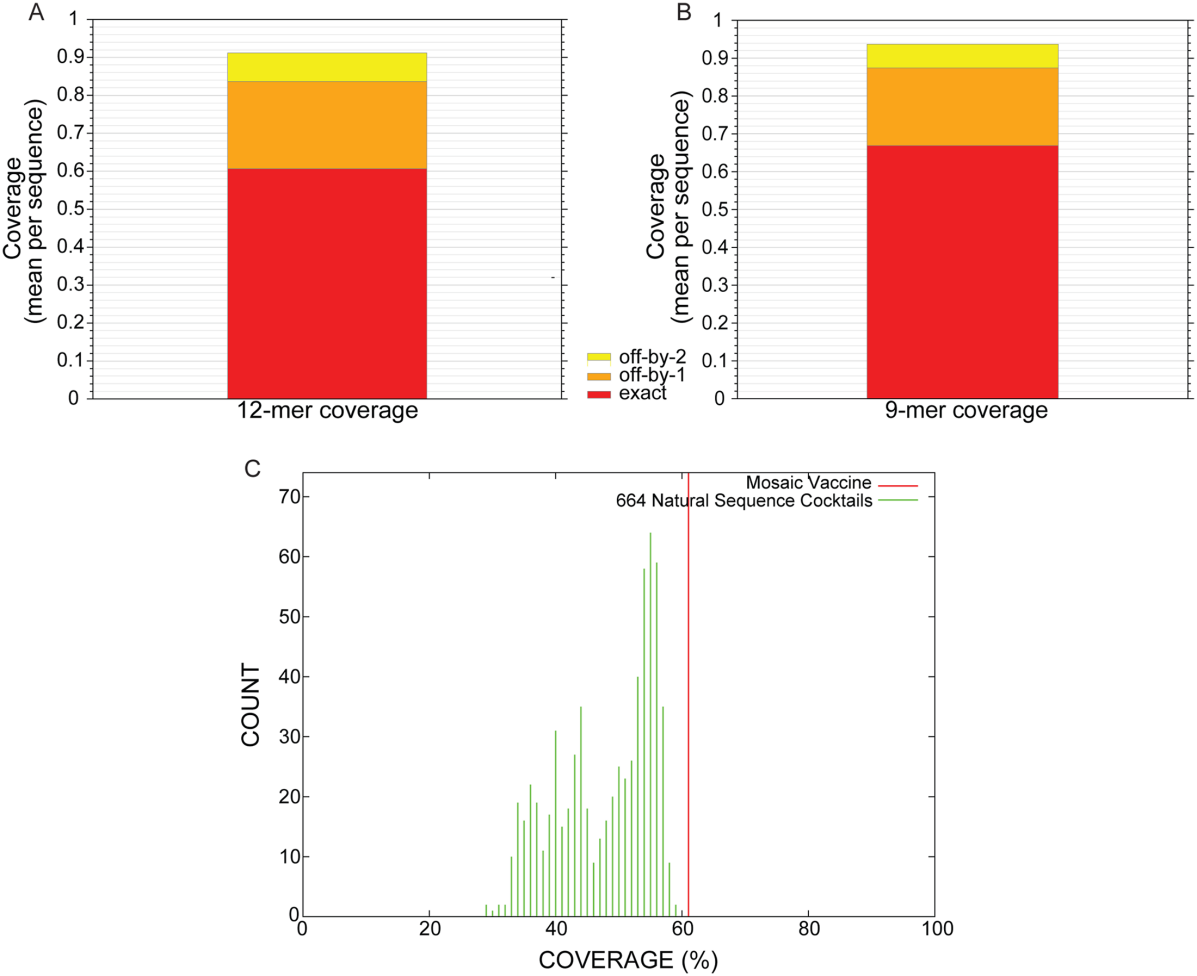
Antigenic coverage of putative T cell epitopes by the designed mosaic phylogroup I lyssavirus glycoprotein. A) Antigenic coverage with the epitope length set to 12 amino acids. B) Antigenic coverage with the epitope length set to 9 amino acids. C) Comparison of 12-mer epitope coverage between the mosaic sequence and all input sequences.

The antigenic coverage of the designed MoG sequence (S1) achieves 61% exact matches of putative T cell epitopes with an epitope length set to 12 amino acids (Fig 1A). This improves to 84% matches if 1 of those 12aa is allowed to be a mismatch (off-by-1) and 92% for off-by-2. This is similar to the results for previously described, effective mosaic proteins[43,44]. If the nominal epitope length is set to 9 amino acids, the coverage increases to 67% exact matches; 87% off by 1; 94% off by 2 (Fig 1B). Comparing epitope coverage of the MoG to the other PG-I lyssaviruses used for its design, it is better than any single "wild type" virus (Fig 1C). A comparison of amino acid sequences of four major and one minor PG-I antigenic sites reveal that MoG retains most RABV sequences (Table 1).

**Table 1:** Amino acid sequence of major phylogroup I lyssavirus antigenic sites based on Evans et al. 2012[45], including mosaic G. The underlined residues are those that differ from the RABV sequence, given in the top row.

Virus	Site IIb (34–42)	Site IIa (198–200)	Site I (226–231)	Site IV (263–264)	Site III (330–338)	Site ‘a’ (342–343)
RABV	GCTNLSEFS	KRA	KLCGVL	FH	KSVRTWNEI	KG
ABLV	GCTSLSGFS	KKA	KLCGIS	FN	KSVRTWDEI	KG
ARAV	GCTNLSGFT	KKA	KLCGVM	FH	KSVREWTEV	KG
BBLV	GCTTLTVFS	KKA	KLCGVS	FH	KSIRQWTEI	KG
DUVV	GCTTLTPFS	KKA	RLCGIS	FH	KSVREWKEI	KG
EBLV-1	GCTTLTPFS	KKA	RLCGVP	FH	KSVREWKEV	KG
EBLV-2	GCTTLTVFS	KKA	KLCGIS	FH	KSIREWTDV	KG
IRKV	GCTTLTAFN	KKA	KLCGMA	DR	KSIREWKEI	KG
KHUV	GCTTLSGFT	KKA	KLCGVS	FH	KSIREWSEI	KG
MoG	GCTNLSGFS	KRA	KLCGVL	FH	KSVRTWNEI	KG

**Fig 2:**
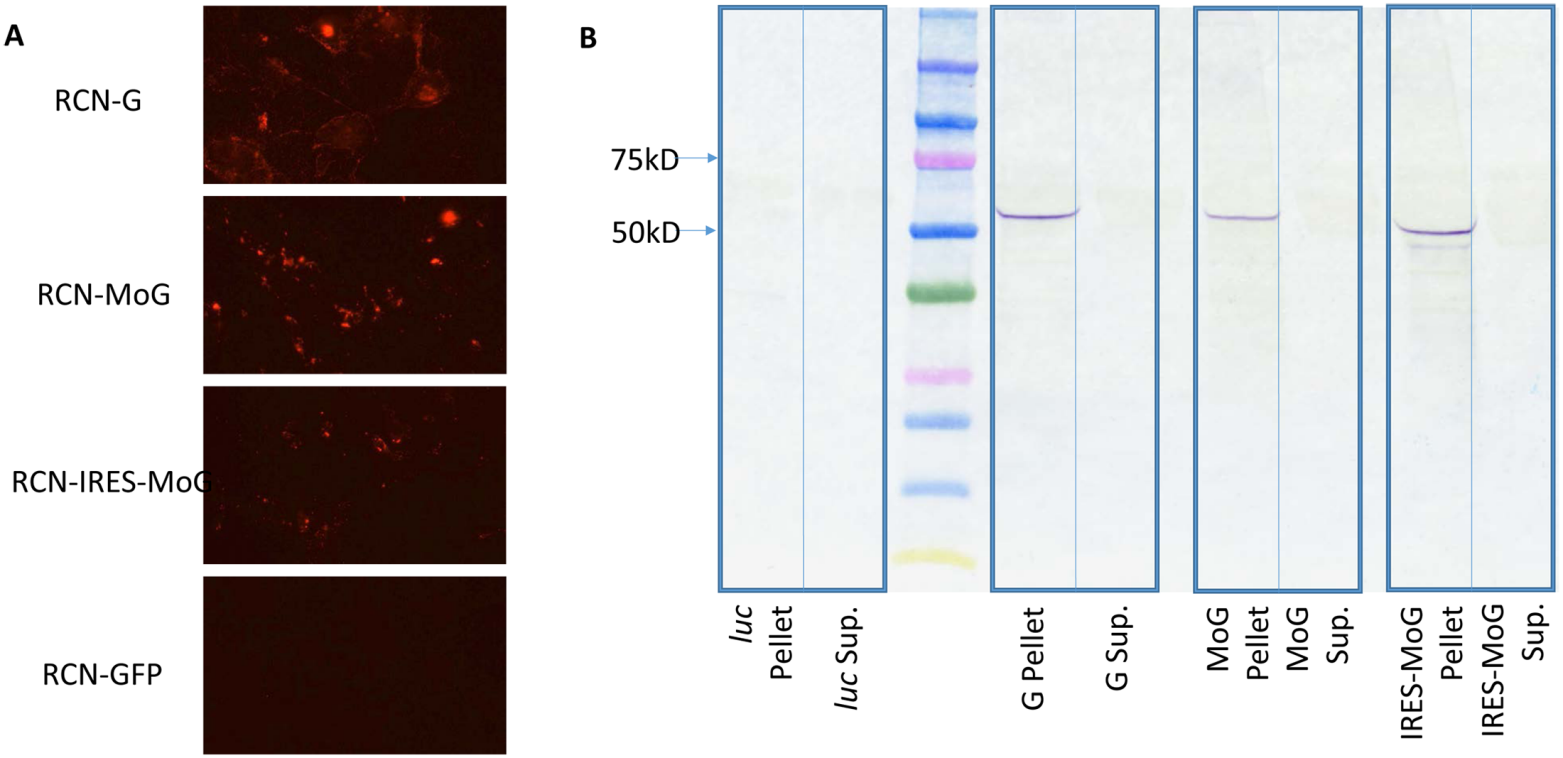
*In vitro* assessment of rabies glycoprotein expression in novel RCN-vectored rabies vaccines. A) Immunofluorescence of RCN expressing *in silico* designed lyssavirus phylogroup I glycoprotein (MoG) with and without an internal ribosomal entry site (IRES). A previously described RCN construct expressing the glycoprotein from rabies CVS-11 (RCN-G) was used as a positive control, and RCN expressing green fluorescent protein (GFP) was used as a negative control. B) Western blot of supernatant (Sup.) or pellet collected from Vero cells infected with RCN-MoG, RCN-IRES-MoG, RCN-G (positive control) or RCN-*luc* (negative control), The rabies glycoprotein is expected to be around 62 kDa.

Immunofluorescence assays of cultured cells infected with RCN-MoG, RCN-IRES-MoG, and RCN-G confirmed presence of rabies virus antigen when compared to the RCN-GFP negative control (Fig 2A). Western blot analysis revealed bands visible at ~60kDa in the pellet of the RCN-MoG, RCN-IRES-MoG, and RCN-G infected cells, and absent in the negative control, demonstrating expression of an antigenic glycoprotein (Fig 2B). The RCN-IRES-MoG seems to be slightly smaller and have a secondary band, which may indicate variation in glycosylation [46] or production of truncated forms of the MoG.

### Immunogenicity of RCN-vectored MoG vaccines and survival upon challenge in mice

**Fig 3:**
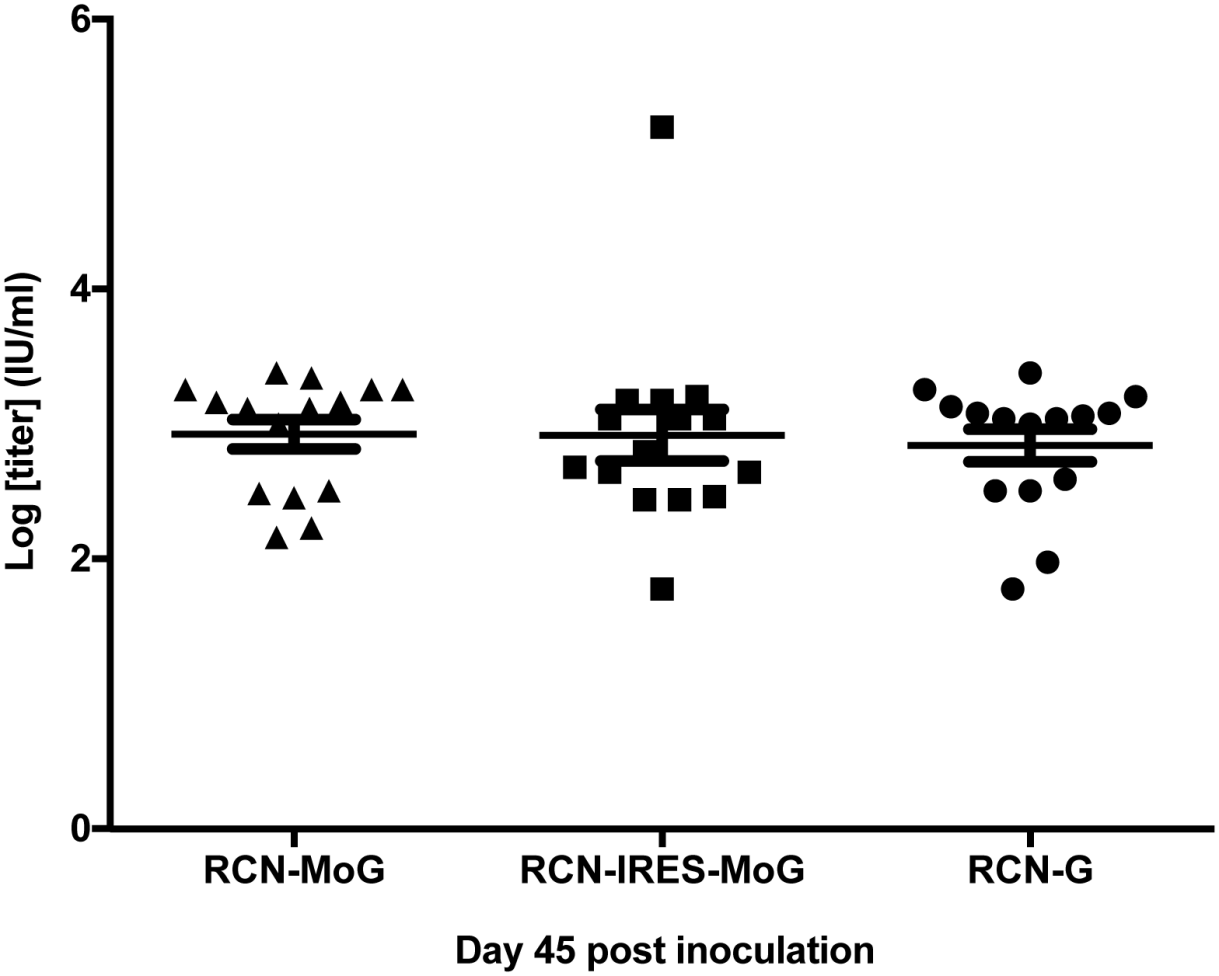
Rabies neutralizing antibody levels in mice following vaccination with various RCN-vectored rabies vaccines. Serum titers of rabies neutralizing antibodies (IU/ml) in mice 45 days post vaccination with RCN-MoG, RCN-IRES-MoG, or RCN-G. No significant differences were detected between groups (P = 0.399).

Serum samples from mice (n = 16 per group) were tested by the RFFIT assay (at CDC). All RCN-rabies constructs induced significant antibody titers when measured at 45 dpv (Fig 3). No significant differences in antibody levels were observed between groups (P = 0.399).

**Fig 4:**
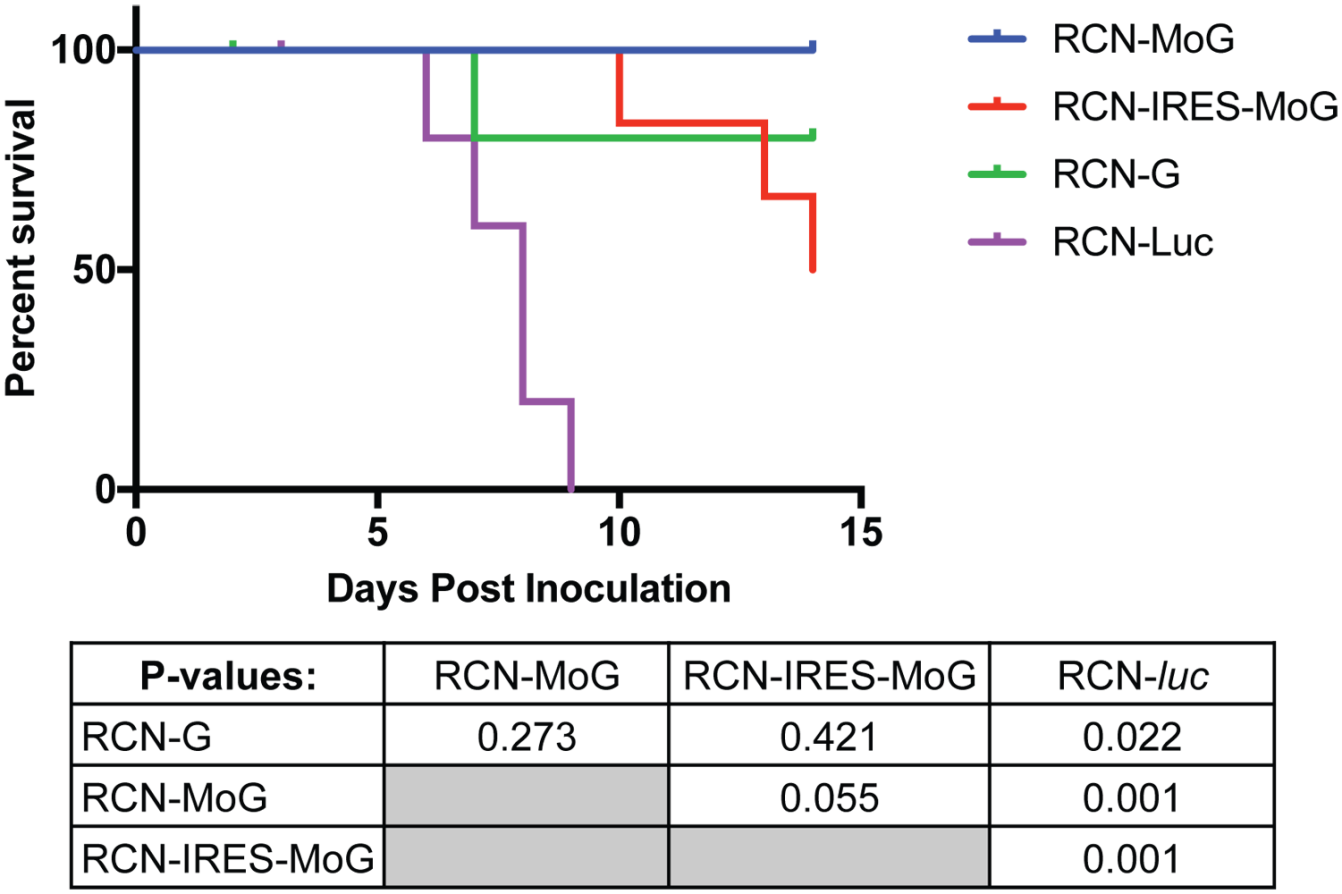
Survival after rabies challenge in mice. Efficacies of raccoon poxvirus (RCN) vectored rabies vaccines in mice after intracerebral challenge with the CVS-11 strain of rabies virus. Every mouse (6/6) in the RCN-MoG group survived challenge to day 14 compared to 3 of 6 in the RCN-IRES-MoG group, and 4 of 5 in the RCN-G group. All (5/5) negative controls (RCN-*luc*) succumbed by day 9 post challenge. A chart of p-values associated with the survival curve is also provided. Survival of all vaccinated mice was significantly higher (P < 0.05) than negative controls, but there was no significant difference (P > 0.05) between vaccine treated groups.

Following rabies virus challenge, one mouse each from the RCN-G and RCN-*luc* groups were euthanized due to loss of ≥ 20% of their body weight within 3 days post challenge (dpc; Table 2). Two other mice, one in each of the RCN-G and RCN-IRES-MoG groups were found to have lost ≥ 20% of their body weight by14 dpc, the last day of the trial. All four of these mice were rabies negative by the dFA test (Table 2) and were censored in the survival analysis. All other mice that were euthanized with signs of disease during the challenge were positive by dFA. All RCN-rabies treatment groups had statistically higher survival than the RCN-*luc* negative controls (P<0.03). All mice survived to day 14 in the RCN-MoG group compared to 50% (3/6) in the RCN-IRES-MoG group, 80% (4/5) in the RCN-G group and 0/5 in the RCN-luc group. Although no significant difference (P >0.05) in survival was detected between groups that received the three rabies vaccines (Fig 4), RCN-IRES-MoG was not included in further studies in bats.

**Table 2:** Survival and % change in weight of vaccinated mice prior to and following challenge with rabies virus.

Group	Mouse ID	Day of death or euthanasia	% weight change	Challenge outcome	dFA rabies diagnosis
RCN-MOG	1		1.04	Survived	Negative
	2		1.02	Survived	Negative
	3		1.03	Survived	Negative
	4		1.00	Survived	Negative
	6		0.99	Survived	Negative
	8		0.97	Survived	Negative
RCN-IRES MOG	1	14	0.79	Died	Positive
	2		1.02	Survived	Negative
	3	13	0.79	Died	Positive
	4	10	0.77	Died	Positive
	5		0.90	Survived	Negative
	6	14	0.77	Censored	Negative
RCN-G	1	8	0.77	Died	Positive
	3		1.00	Survived	Negative
	4		0.92	Survived	Negative
	5	3	0.68	Censored	Negative
	6		1.06	Survived	Negative
	7	14	0.79	Censored	Negative
RCN-luc	9	9	0.80	Died	Positive
	10	3	0.80	Censored	Negative
	11	7	0.80	Died	Positive
	13	8	0.75	Died	Positive
	14	8	0.76	Died	Positive
	15	6	0.77	Died	Positive

### Immunogenicity of RCN-vectored vaccines and survival upon challenge in bats

After inoculation with RCN-vectored vaccines, no signs of clinical disease were evident in any of the bats. Topically vaccinated bats also showed no evidence of adverse effects due to the glycerin jelly application or the vaccine virus. No significant change in weight was evident in the groups after initial vaccination or boost (P>0.05, S1 Fig).

**Fig 5:**
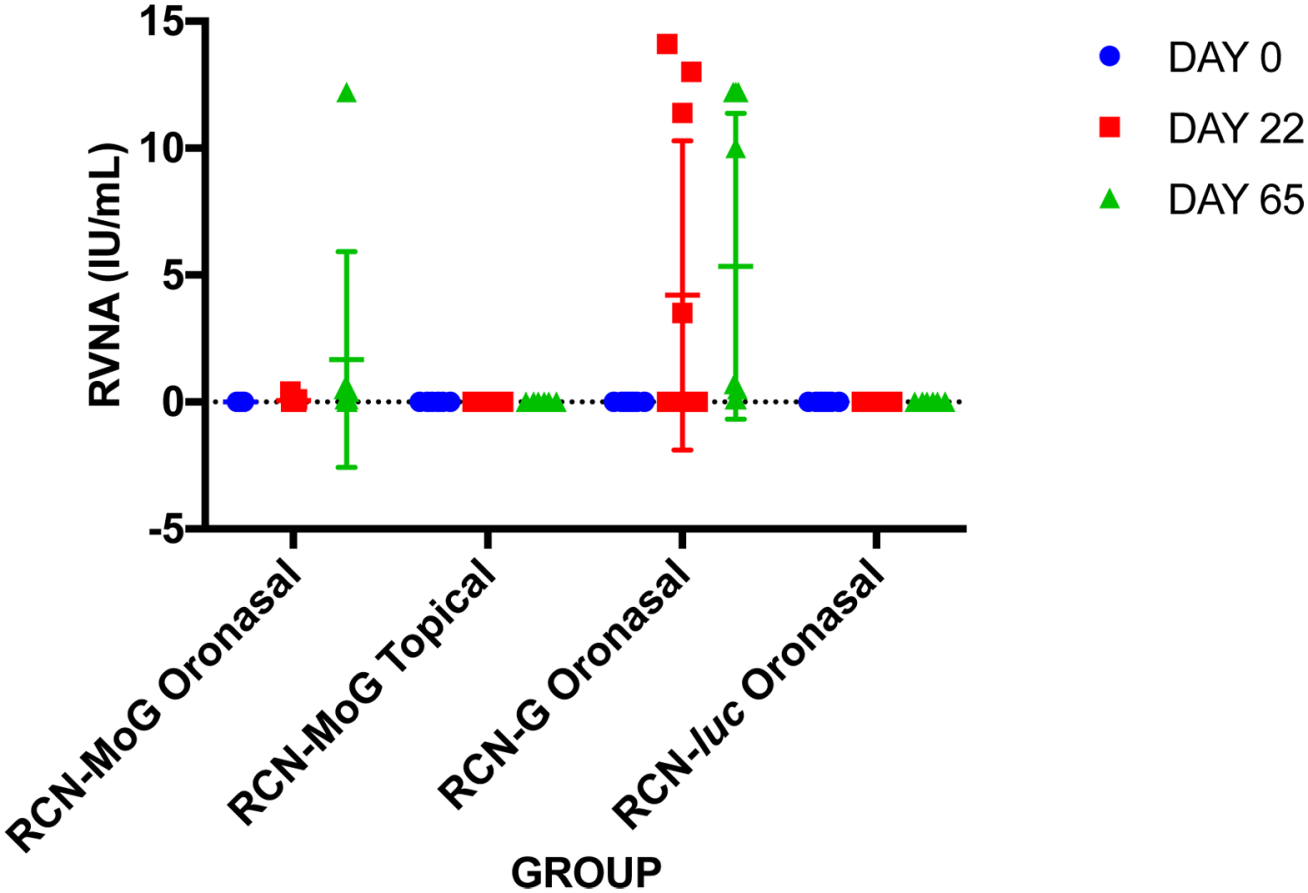
Rabies virus neutralizing antibodies in bats following oronasal vaccination with RCN-based rabies vaccine constructs. Serum rabies neutralizing antibody titers at various time-points as determined by rapid fluorescence focus inhibition test (RFFIT). Day 22 represents levels after initial vaccination, and day 65 represents levels after boost and immediately prior to challenge.

After initial vaccination, 2/9 bats from the RCN-MoG ON group had titers between 0.1–0.4 IU/ml and 4/10 bats from the RCN-G ON group responded with titers >0.5 IU/ml, while no detectable antibodies were found in any of the bats from the RCN-*luc* group or the RCN-MoG topically vaccinated bats (Table 3, Fig 5). After boost, 2/8 bats tested in the RCN-MoG ON group had titers > 0.5 IU/ml, and an additional 4 bats had titers of 0.1–0.4. In the RCN-G ON group, 6/10 bats had RVNA levels ≥ 0.5 IU/ml, 3 had levels of 0.1–0.4, and one bat had no detectable RVNA. Even though more bats that received RCN-G ON had RVNA titers compared to RCN-MoG ON, no significant difference in titer was detected between these groups (P = 0.22). Bats in the RCN-*luc* and RCN-MoG topically vaccinated groups had no detectable neutralizing antibodies prior to challenge.

**Table 3:** Serological and survival results of vaccinated *E*. *fuscus* prior to and following challenge with rabies virus.

Group	Bat ID	Rabies virus titer VNA (IU/ml) days post primary inoculation		Challenge outcome	dFA rabies diagnosis
		22	65		
RCN-MoG (Oronasal)	1504	0.0	0.1	Survived	Negative
	1511	0.0	0.1	Survived	Negative
	1518	0.0	0.5	Survived	Negative
	1525	0.1	0.4	Survived	Negative
	1526	0.0	0.0	Survived	Negative
	1528	0.0	0.0	Survived	Negative
	1530	0.4	12.2	Survived	Negative
	1531	0.0	no sample	Survived	Negative
	1535	0.0	0.1	Survived	Negative
RCN-MoG (Topical)	1512	0.0	0.0	Survived	Negative
	1523	0.0	0.0	Survived	Negative
	1527	0.0	0.0	Survived	Negative
	1529	0.0	0.0	Died	Positive
	1536	0.0	0.0	Survived	Negative
	1537	0.0	0.0	Survived	Negative
RCN-G (Oronasal)	1506	13.0	12.2	Survived	Negative
	1507	3.5	12.2	Survived	Negative
	1508	0.0	0.1	Survived	Negative
	1517	0.0	0.1	Died	Positive
	1519	11.4	10.0	Survived	Negative
	1520	0.0	0.0	Died	Positive
	1522	0.0	0.5	Died	Positive
	1524	0.0	0.7	Survived	Negative
	1532	14.1	12.2	Survived	Negative
	1534	0.0	0.1	Survived	Negative
RCN-luc (Oronasal)	1501	0.0	0.0	Died	Positive
	1509	0.0	0.0	Died	Positive
	1513	0.0	0.0	Died	Positive
	1514	0.0	0.0	Survived	Negative
	1515	0.0	0.0	Died	Positive
	1521	0.0	0.0	Died	Positive
	1533	0.0	0.0	Died	Positive
	1538	0.0	0.0	Died	Positive

**Fig 6:**
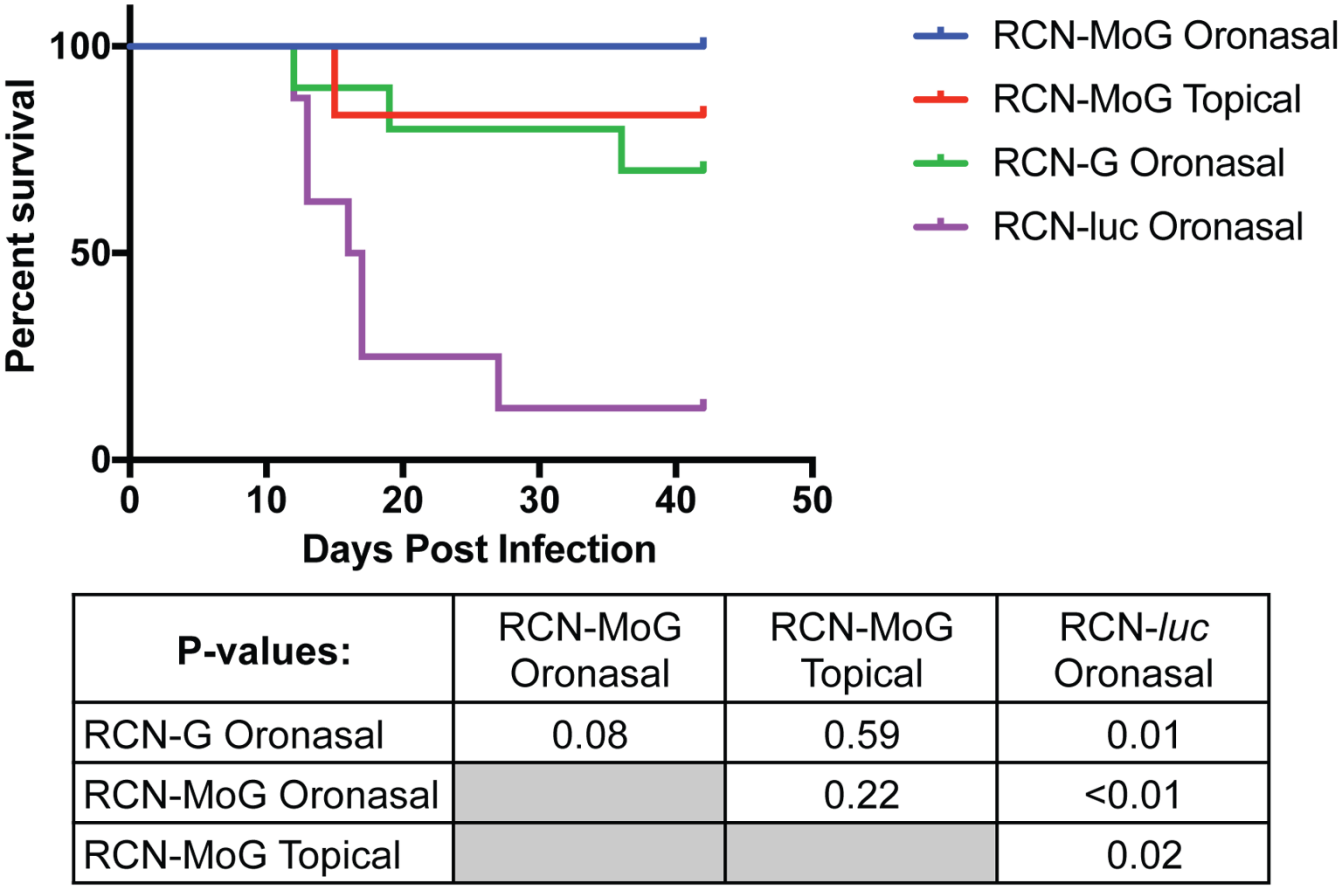
Survival after rabies challenge in *E*. *fuscus* bats. Percent survival of *E*. *fuscus* bats is shown over time after experimental infection. Bats were vaccinated oronasally with RCN-MoG, RCN-G, or RCN-l*uc* (negative control). A fourth group was given RCN-MoG topically in a glycerin jelly vehicle. Vaccinated bats had significantly greater survival than negative controls (P = 0.002).

After challenge with rabies virus, all vaccine treatment groups had significantly greater (P ≤ 0.02) rates of survival than the negative control (RCN-*luc*) group (Fig 6). The first confirmed rabies deaths occurred at 12 dpc and the final at 27 dpc. The majority of mortalities occurred between 12 and 19 dpc. All bats administered RCN-MoG by the ON route survived challenge, although interestingly only 2/8 had pre-challenge RVNA levels above 0.5 IU/ml (Table 2, Fig 5). Likewise, 5/6 (83%) of the bats that received RCN-MoG topically survived challenge, despite none having seroconverted. Comparatively, 7/10 of the RCN-G ON vaccinated group survived challenge, including two bats with antibody titers below 0.5 IU/ml. Interestingly, one bat in this group with a titer of 0.5 IU/ml succumbed to rabies challenge and 1/8 bats immunized with RCN-*luc* survived challenge.

No clinical signs were observed in any of the surviving bats. Direct FA confirmed rabies diagnoses consistent with our survival analysis (Table 2). All bats that were found dead or euthanized were rabies positive, while all remaining bats at the study end were negative.

## Discussion

Rabies spillover from wildlife, particularly by vampire bats (*Desmodus rotundus*), continues to be an important public health and economic issue in Mexico and Central and Latin America [17,47], despite using culling of bats as a control measure[48–50]. In this study, we demonstrated that an *in silico* designed mosaic lyssavirus PG-I glycoprotein (MoG) is an effective immunogen against rabies in mice and bats. Furthermore, a recombinant RCN-vectored vaccine expressing MoG, delivered by mucosal or topical routes, protected bats against rabies challenge. While survival did not differ significantly among any of the vaccine treated groups (P = 0.08), RCN-MoG provided 100% protection in ON immunized bats challenged with a wild-type big brown bat RABV variant. As in our previous study [22], both RCN vaccine constructs were safe; no evidence of morbidity was observed in treated bats. Though these results are very promising, additional challenge studies with other bat RABV variants, are needed to assess whether our bioinformatically designed RCN-MoG vaccine is an improvement over RCN-G.

Currently available rabies vaccines, which are almost entirely developed from lab-adapted strains (e.g. CVS-11), are considered protective against all PG-I lyssaviruses when given at the recommended dose and schedule. However, antigenic variation in PG-I strains has been identified and may lead to inconsistent protection [12,13]. The CVS-11 strain has been passaged over a thousand times in rabbit and mouse brains and cell culture [51]. One study showed that 5.1 units of antigenic difference exists between CVS-11 and “wild type” RABV strains isolated from different hosts, equivalent to a more than 10-fold dilution in antibody titer[12]; thus higher titers are needed for protection. For wildlife consuming variable doses of vaccines via the oral route of delivery, it is important to use the most efficient vaccine, protective at the lowest titer possible with the fewest doses, as boosts are generally unfeasible. Although bats were boosted in our initial study to optimize their response, testing of a single dose application will be critical in future studies.

In an attempt to maximize vaccine efficiency, we designed MoG to be more broadly representative of all PG-I lyssavirus glycoproteins. MoG has 93% similarity to the wild-type big brown bat variant RABV used in the challenge study. The glycoprotein of the CVS-11 strain has 94.7% consensus amino acid similarity to MoG, but only 90% similarity to the big brown bat variant RABV. The higher level of similarity between MoG and the challenge strain, as compared to the CVS-11 G protein, may have resulted in the slightly higher survival of RCN-MoG vaccinated bats (survival 9/9) compared to RCN-G vaccinated bats (survival 7/10), although the difference observed between these small groups was not statistically significant.

Mosaic proteins are synthetically designed to represent all potential epitopes from related input sequences and have been shown to induce greater cross-reactivity than consensus sequences [52]. Thus, we expected the immune response elicited by vaccination with MoG to be more efficient at neutralizing naturally circulating RABV than current antigens, however this was not detected by RFFIT (Table 2). Interestingly, RVNA did not correlate directly with survival. Specifically, topically vaccinated bats, as well as some bats vaccinated ON with RCN-MoG, did not seroconvert prior to challenge, yet survived. While it is generally believed that RVNA are needed for protection, results similar to ours have been reported elsewhere [53–56]. In our case, it is possible that the RCN-MoG vaccine may be better at priming T_H_ cells or activating other adaptive cellular immune responses necessary for clearance of RABV [57–61]. The use of viral vaccine vectors usually leads to a Th1, CTL response directed at the target antigen. The earlier production of antigen due to the S E/L promoter also leads to an increased CTL response[62]. It is possible that CD8 cells, elicited by vaccination with RCN-MoG, lysed infected cells shortly after challenge, resulting in protection in the absence of detectable neutralizing antibody responses. The enhanced inflammatory response induced by activated CD8 T cells may also have contributed to antibody-mediated clearance, as has been previously suggested[60]. In follow-up studies, it would be useful to assess the cellular immune response to vaccination.

Alternatively, it is possible that RVNA induced by RCN-MoG were not properly recognized due to the use of CVS-11 strain in the RFFIT analysis. Thus, it might be necessary to develop a RIFFT assay with MoG as the substrate antigen and to compare the neutralizing capacity of antibodies induced by both RCN-MoG and RCN-G constructs to various divergent lyssaviruses.

The studies presented here are especially relevant for vampire bats. So far, most efforts to reduce their threat have centered on culling through the application of anticoagulants to individual bats that are released to poison additional bats through contact and commensal grooming. Vampire bats in particular are known to practice self and social grooming at a very high rate [63], so this method of application is very effective. Unfortunately, culling of bats has largely failed to reduce the incidence of bovine rabies and may be counterproductive for disease control [49,50,64]. Also, this method frequently leads to indiscriminate killing of other bat species [3], which are key members of their ecosystems. Instead, by immunizing certain vampire bat populations against rabies with sufficient coverage to create herd immunity, it may be possible to reduce rabies transmission, thereby lowering the risk of exposure to humans and livestock.

Previous laboratory studies have demonstrated successful topical vaccination of *Desmodus* using a vaccinia virus expressing the glycoprotein from the ERA strain of rabies (VR-G) [53,65,66]. However, the vaccinia vector can infect humans, especially immunocompromised individuals [18,67], and oral delivery of this vaccine to vampire bats induced lower levels of rabies neutralizing antibodies than oral delivery of RCN-G to *E*. *fuscus* in this study and *T*. *brasiliensis* in our previous study [22]. With further testing in vampire bats, RCN-MoG may offer a safer, more effective alternative that could be delivered topically via glycerin jelly or another medium. For a topical vaccine to be practical and effective, it must induce significant immunity after limited oral exposure and must be applied in an appropriate medium that maintains vaccine titer for extended periods in ambient conditions and attaches firmly to the fur of the target species. Although glycerin jelly was effective in our initial studies, more work is required to determine its utility as a delivery medium for free-ranging bats. An alternative to topical application of vaccine may be aerosolized application to roost sites in caves, but that remains to be tested.

Finally, this approach could be adapted for other species or groups of bats and for other important diseases, such as white nose syndrome, a fungal disease killing millions of bats in North America [68]. While much effort has gone into identifying and characterizing the pathogens carried by bats, little has been done to prevent disease in bat hosts. Successful vaccination of bats against rabies could potentially lead to the development of other bat-targeted vaccines.

## Supporting information

S1 Fig Bat weights over time. Bat weights (in g with SD) are shown over time (each date as a time-point), with vertical bars denoting the date of initial vaccination (1/25), boost dose (3/10), and rabies challenge (3/29). No significant weight loss is appreciable after vaccination with RCN constructs.
(TIFF)
