# Supplementary figures and images for: Protection of bats (Eptesicus fuscus) against rabies following topical or oronasal exposure to a recombinant raccoon poxvirus vaccine
Source: PLoS Negl Trop Dis. 2017 Oct 4;11(10):e0005958. doi: 10.1371/journal.pntd.0005958 (PMC5643138; doi:10.1371/journal.pntd.0005958)

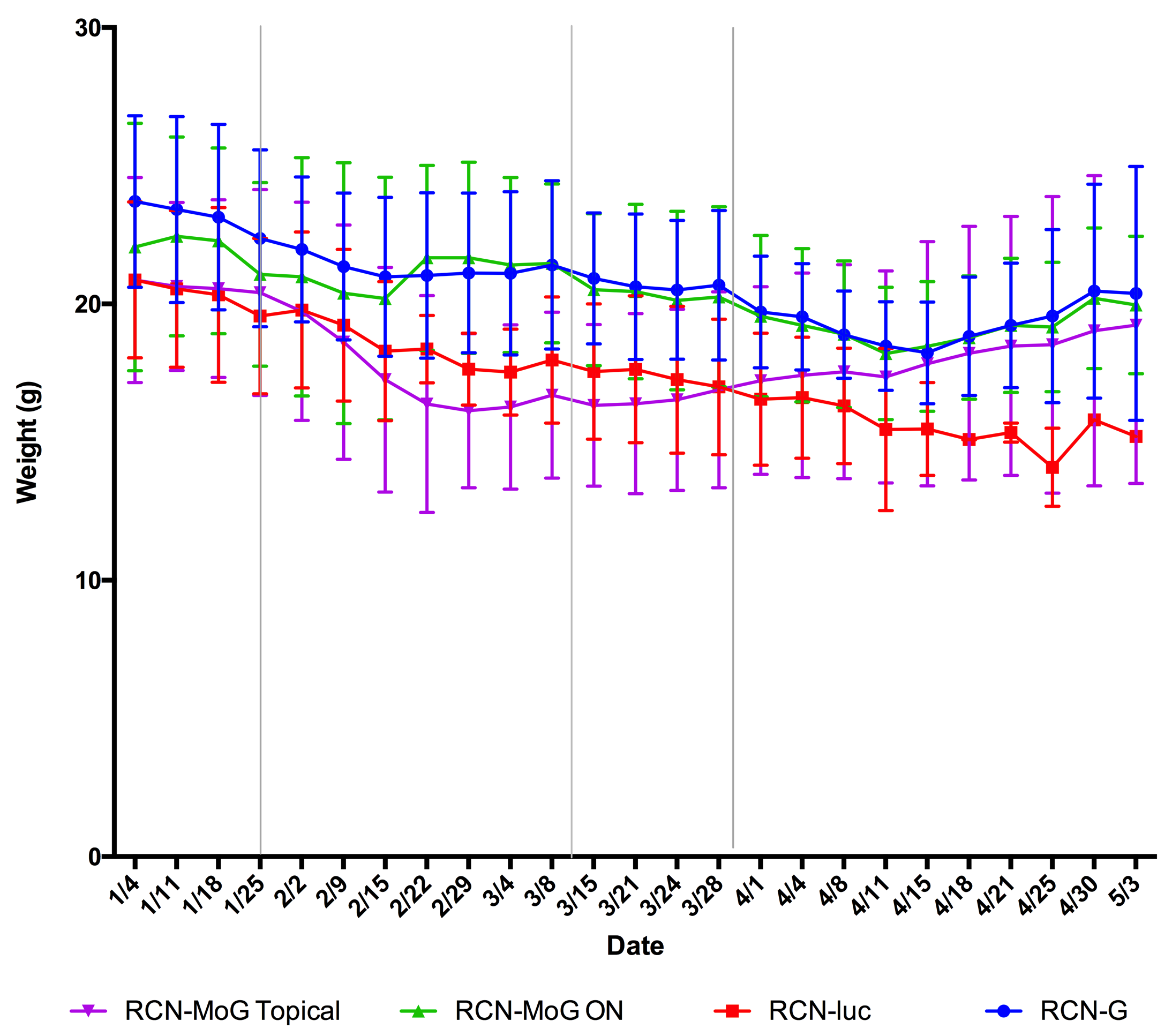

Supplement: S1 Fig — Bat weights (in g with SD) are shown over time (each date as a time-point), with vertical bars denoting the date of initial vaccination (1/25), boost dose (3/10), and rabies challenge (3/29). No significant weight loss is appreciable after vaccination with RCN constructs. (TIFF) [file pntd.0005958.s001.tiff]
